# Pathology of Synapses and Dendritic Spines

**DOI:** 10.1155/2012/972432

**Published:** 2012-09-23

**Authors:** Shira Knafo, Gunnar K. Gouras, Xiao-Xin Yan, Tara Spires-Jones

**Affiliations:** ^1^Centro de Biología Molecular “Severo Ochoa,” Consejo Superior de Investigaciones Científicas (CSIC) and Universidad Autónoma de Madrid, Nicolás Cabrera, 28049 Madrid, Spain; ^2^Experimental Dementia Research Unit, Wallenberg Neuroscience Center and Department of Experimental Medical Science, Lund University, Sölvegatan 19, 221 84 Lund, Sweden; ^3^Department of Anatomy and Neurobiology, Central South University Xiangya School of Medicine, Changsha, Hunan 410013, China; ^4^Massachusetts General Hospital, Harvard Medical School, 114 16th Street, Charlestown, MA 02129, USA

Excitatory synapses represent the sites in which axons make a functional contact with their target neurons and they are typically located at the head of dendritic spines. Due to their critical role as mediators of interneuronal interactions, insults to synapses may result with significant clinical manifestations such as dementia or movement disorders. This special issue of Neural Plasticity discusses various aspects of synapse and spine pathology associated with Alzheimer's disease, perinatal asphyxia, and neuropathic pain.

Synapse and spine loss may represent early and profound neuropathological changes potentially underlying cognitive deficits in Alzheimer's disease (AD). In fact, synapse loss is the strongest pathological correlate of dementia in AD. The inherent plasticity of synapses makes them an attractive therapeutic target. W. Yu and B. Lu, “*Synapses and dendritic spines as pathogenic targets in Alzheimer's disease,*” review the important field of synapse and dendritic loss in AD. In their paper, the authors discuss the well-established role of oligomeric amyloid beta in causing synaptic dysfunction and loss through signaling mechanisms associated with long-term depression. They also explore the exciting new relationship between amyloid beta and tau at the postsynaptic density. Several recent studies have placed tau in dendritic spines—a surprising place for a protein that dogma places firmly on microtubules in axons—where it is turning out to mediate amyloid-beta-induced synaptic dysfunction and loss.

In another review T. Spires-Jones and S. Knafo, “*Spines, plasticity, and cognition in Alzheimer's model mice,*” provide a comprehensive analysis on recent works describing the morphological, synaptic, and behavioral characteristics of the different transgenic models of AD. Results from various models and in variety of ages show a gradual deterioration in synaptic and cognitive functions. The accumulating evidence from transgenic models discussed in this review appears to support a model of AD pathogenesis in which oligomeric amyloid beta initiates synaptic dysfunction or degeneration and induces pathological changes in tau, leading to neuronal loss and ultimately to cognitive deficits. 

G. E. Saraceno et al., “*Hippocampal dendritic spines modifications induced by perinatal asphyxia,*” investigated the effect of perinatal asphyxia (PA) on the hippocampal postsynaptic density (PSD). They report an unexpected increased thickness and dispersed appearance of the PSD in the asphyctic brains. Correlative fluorescent and electron microscopy showed a decline of F-actin-stained spines in hippocampal excitatory synapses after the insult that may suggest that PA is harmful to the actin cytoskeleton. These data suggest that perinatal asphyxia may lead to long-term changes in hippocampal synapses.

NMDA receptors are located at synapses and modulate various forms of synaptic plasticity. Grin1b gene encodes the postsynaptic NMDA receptor in zebrafish. RNA and various RNA products play critical roles in regulating gene expression and production of neuroactive proteins in the nervous system. P. Pozo and B. Hoopengardner, “*Identification and characterization of two novel RNA editing sites in grin1b transcripts of embryonic Danio rerio,*” identified two novel RNA editing events for the grin1b gene in zebrafish. This new information may have implications for transcriptional regulation of mammalian glutamate receptors, which play essential roles in neuronal transmission and plasticity, but also mediate neuronal toxicity in neurodegenerative disorders.

S. K. Kim et al., “*Synaptic structure and function in the mouse somatosensory cortex during chronic pain: in vivo two-photon imaging,*” summarize recent developments in studying *in vivo* spine dynamics in thesomatosensory cortex of adult mice in a model of chronic neuropathic pain. Thearticle highlights theimportance of neural plasticity in pain research. Employingmultiphoton microscopy, they describe remarkably rapid synaptic remodeling inlayer 5 neurons of somatosensory cortexwithin days of the peripheral nerveinjury (partial sciatic nerve ligation). Peripheral nerve injury via peripheralhyperactivity causes a rapid rewiring of distinct somatosensory cortexsynapticconnections, leading to local somatosensory cortex hyperexcitability. Theauthors postulate that these local cortical changes in spine turnover followinginduction of neuropathicpain play an important role in chronic pain conditions.

*Shira Knafo* *Shira Knafo* *Gunnar K. Gouras* *Gunnar K. Gouras* *Xiao-Xin Yan* *Xiao-Xin Yan* *Tara Spires-Jones* *Tara Spires-Jones*

